# Task-shifting directly observed treatment and multidrug-resistant tuberculosis injection administration to lay health workers: stakeholder perceptions in rural Eswatini

**DOI:** 10.1186/s12960-020-00541-4

**Published:** 2020-12-03

**Authors:** Ernest Peresu, J. Christo Heunis, N. Gladys Kigozi, Diana De Graeve

**Affiliations:** 1 Centre for Development Support, Faculty of Economic and Management Sciences, University of the Free State, P.O. Box 399, Bloemfontein, 9300 South Africa; 2 Centre for Health Systems Research & Development, University of the Free State, P.O. Box 399, Bloemfontein, 9300 South Africa; 3 Faculty of Business and Economics, University of Antwerp, Prinsstraat 13, 2000 Antwerp, Belgium

**Keywords:** Eswatini, Human resources for health, MDR-TB, Community treatment supporter, Directly observed treatment, Injection administration

## Abstract

**Background:**

Eswatini is facing a critical shortage of human resources for health (HRH) and limited access to multidrug-resistant tuberculosis (MDR-TB) treatment in rural areas. This study assessed multiple stakeholders’ perceptions of task-shifting directly observed treatment (DOT) supervision and administration of intramuscular MDR-TB injections to lay health workers (LHWs).

**Methods:**

A mixed methods study comprising a cross-sectional survey using a semi-structured questionnaire with community treatment supporters (CTSs) and a focus group discussion with key stakeholders including representatives from the Eswatini Ministry of Health (MOH), donor organisations, professional regulatory institutions, nursing academia, civil society and healthcare providers was conducted in May 2017. Descriptive statistics, thematic content analysis and data triangulation aided in the interpretation of results.

**Results:**

A large majority of CTSs (*n* = 78; 95.1%) were female and 33 (40.2%) were older than 50 years. Most (*n* = 7; 70.0%) key stakeholders had over 10 years of work experience in policy-making, advocacy in the fields of HRH or day-to-day practice in MDR-TB management. Task-shifting of MDR-TB injection administration was implemented without national policy guidance and regulation. Stakeholders viewed the strategy to be driven by the prevailing shortage of professional frontline HRH and limited access to MDR-TB treatment. Task-shifting was perceived to improve medication adherence, and reduce stigma and transport-related MDR-TB treatment access barriers. Frontline healthcare workers and implementing donor partners fully supported task-shifting. Policy-makers and other stakeholders accepted task-shifting conditionally due to fears of poor standards of care related to perceived incompetence of CTSs. Appropriate compensation, adequate training and supervision, and non-financial incentives were suggested to retain CTSs. A holistic task-shifting policy and collaboration between the MOH, academia and nursing council in regulating the practice were recommended.

**Conclusions:**

Stakeholders generally accepted the delegation of DOT supervision and administration of intramuscular MDR-TB injections to LHWs as a strategy to increase access to treatment, albeit with some apprehension. Findings from this study stress that task-shifting is not a panacea for HRH shortages, but a short-term solution that must form part of an overall simultaneous strategy to train, attract and retain adequate numbers of professional healthcare workers in Eswatini. To address some of the apprehension and ambivalence about expanding access to MDR-TB services through task-shifting, attention should be paid to important aspects such as competence-based training, certification and accreditation, adequate supportive on-the-job supervision, recognition, compensation, and expediting policy and regulatory support for LHWs.

## Background

Sub-Saharan Africa is facing the most serious shortage in human resources for health (HRH) in the world. Eswatini is among 57 countries classified as experiencing a critical professional healthcare worker shortage, with 1.40 midwives, nurses and physicians per 1000 population [1]. This is far below the benchmark of 2.28 healthcare workers per 1000 population recommended for adequate coverage of essential health services [2]. In Eswatini, the rural areas carry a disproportionately high burden of multidrug-resistant tuberculosis (MDR-TB) and have primary healthcare (PHC) clinics that are often far and geographically inaccessible from the patient’s home, and are typically also characterised by a lack of frontline TB HRH [3–7]. Frontline TB HRH in this setting refers to health personnel who are usually the community’s first port of call for accessing TB control services.

The control of TB has been highlighted as a priority in the Eswatini National TB Strategic Plan [8], the post-2015 global TB strategy (the End TB Strategy) [9] and the Sustainable Development Goals (SDGs) agendas [10]. In 2017, Eswatini reported a 68% treatment success rate for MDR-TB patients (2015 cohort), which was below the World Health Organization (WHO) target of 75% or higher [3, 11, 12]. The critical shortage of HRH and extent of the MDR-TB epidemic, particularly in rural areas, has seen the number of patients requiring access to MDR-TB treatment exceed the current capacity of the Eswatini Ministry of Health (MOH). The serious HRH scarcity impedes the country’s ability to implement and leverage interventions aimed at achieving national and international MDR-TB control targets.

In order to address the prevailing professional frontline HRH and MDR-TB treatment access challenges in the predominantly rural Shiselweni region, Médecins Sans Frontières (MSF) established a novel patient-centred community-based MDR-TB treatment strategy in 2008 within the existing National Tuberculosis Control Programme (NTCP) [7]. The main feature of this model is the task-shifting of directly observed treatment (DOT) supervision and intramuscular MDR-TB injection administration responsibilities traditionally restricted to professional nurses to trained lay health workers (LHWs). DOT involves a patient taking standard medication under guided supervision from a healthcare worker or trained caregiver. Local supervision of DOT and administration of intramuscular injections by a LHW, referred to as a community treatment supporter (CTS), was introduced to address some of the social and financial difficulties frequently faced by patients in accessing the often more distant centralised clinic-based MDR-TB treatment services [7].

In this model of care, MDR-TB patients discharged from the MDR-TB inpatient hospital are linked to a CTS of their own choice and from their locality. Community MDR-TB nurses facilitate the selection, recruitment and training of CTSs. The recruitment of CTSs, which starts as soon as the diagnosis of MDR-TB is made, involves the patient proposing possible neighbours to the community MDR-TB nurse who then selects and contacts the most suitable candidate. The selected neighbour must have sufficient literacy skills to be able to read the community MDR-TB management training manual in English and document treatment [7]. In addition, the CTS should have a stable living accommodation near the patient to perform twice-daily DOT and administer injection-based medication; be available throughout the day and night to support the patient; and be motivated to support the MDR-TB patient for the full length of the treatment.

The training of CTSs comprises of a 5-day theoretical workshop and on-the-job learning. The theoretical component of the training is conducted by community MDR-TB nurses through classroom instruction, group work and open discussions, and focuses primarily on themes relating to MDR-TB epidemiology, transmission, diagnosis, treatment/strategies (DOT), safe injection handling and adverse drug reactions. CTSs also learn about maintaining their patients’ confidentiality during the pre-service training. The hands-on training covers the delicate skill of drawing the right dose of the injectable drug using a disposable syringe, TB infection prevention and control (IPC), DOT and waste disposal. CTSs practise safe injection techniques on an orange fruit before progressing to their recruited client.

Instead of making trips to the nearest clinic, this model of care allows MDR-TB patients to receive DOT and daily injections from CTSs in their (the patients’) homes. CTSs accompany their MDR-TB patients to the main health facilities for their monthly outpatient treatment review or, in the case of worsening health condition, unscheduled visits. CTSs are also expected to identify and promptly report missed doses, possible side effects and medication running out to the community MDR-TB nurse using a mobile phone. CTSs assist the patient in producing sputum samples for monthly MDR-TB laboratory monitoring. In addition, they provide health education and emotional support to the patient and family. Due to the possible risk of HIV exposure from needlestick injuries, newly recruited CTSs are routinely informed of the patient’s HIV status prior to undergoing training. They are also expected to support their patients in antiretroviral medication adherence.

CTSs are directly supervised by community MDR-TB nurses. Using mobile phones, CTSs are able to interact with and receive ‘just-in-time’ supervision support or technical assistance from their supervisors. The community MDR-TB nurses are supported by a team of facility-based professional healthcare workers made up of a doctor, psychologist and social worker. Core clinical decisions such as initiation, variation and monitoring of MDR-TB treatment and progress are taken by formal facility-based professional healthcare workers. CTSs receive a monetary incentive of ZAR500 (= US$33.54 [exchange rate on 23/11/2017]) per month − approximately 5% of the monthly salary community MDR-TB nurses receive − during the 8-month-long injection phase of their patient’s treatment. The monthly incentive is meant to cover out-of-pocket transport expenses CTSs incur when accompanying their patients for outpatient medical reviews. MSF has consistently sustained the provision of this financial incentive to CTSs since the inception of the task-shifting strategy.

Task-shifting has been widely endorsed globally as a remedy for increasing access to healthcare services and professional healthcare worker shortages. Task-shifting is defined as "the rational redistribution of tasks among health workforce teams", and the WHO added in global guidelines issued in 2008 that “specific tasks are moved, where appropriate, from highly qualified healthcare workers to health workers who have fewer qualifications in order to make more efficient use of the available HRH” [13]. More recently, the WHO provided the Global Strategy on Human Resources for Health: Workforce 2030 supporting the use of sustainable, contextually appropriate and cost-effective interventions to address the shortage of HRH particularly in resource-limited settings [14]. The WHO global recommendations and guidelines on task-shifting and previous research have established that the long-term success of the strategy hinges on investment in policy, regulation, pre-service and on-going education, accreditation and certification programmes, reorganisation of health teams, clear scopes of practice, remuneration and simplified guidelines [13, 15–19].

Although the use of task-shifting as a strategy to expand access to health services is most notably credited with the resurgence of LHWs, the approach is accompanied by a unique set of benefits and challenges. Previous research has shown that task-shifting can reduce overhead costs, expand access to care, improve patient experience and optimise programme effectiveness [15–17, 20–22]. However, important concerns remain over whether or not task-shifting to LHWs does not overlook issues related to patient safety and quality of care, accountability, medical liability (if adverse events arise), the ability of lay health workers with limited levels of training to handle complex cases requiring clinical skills of a curative nature, and alignment with wider health system strengthening [17–20, 22].

Despite the existence of the task-shifting strategy for over a decade in the Shiselweni region, the MOH has not yet officially endorsed the strategy. In the absence of a national regulatory policy on task-shifting, MSF implemented the task-shifting strategy after informal permission was granted by the MOH. This raises questions about the perceptions of policy-makers on the task-shifting strategy and whether or not the practice has been implemented in compliance with the WHO recommendations and guidelines. The MOH and professional bodies have raised ethical concerns about standards of care and safety risks for patients and the newly 'skilled-up' LHWs. As a result, there are fears that task-shifting community-based care to CTSs may create a two-tiered system of MDR-TB management, with “superior” and “inferior” tracks [23]. Existing NTCP strategic plans and MDR-TB treatment guidelines only provide for task-shifting of certain professional nurses’ responsibilities such as DOT supervision and TB treatment adherence support to family members, neighbours or former patients.

Although the Shiselweni region has relied largely on task-shifting in expanding MDR-TB treatment access for over a decade, surprisingly, the opinions about the practice of stakeholders in Eswatini are purely anecdotal, understudied and still to be empirically examined. It is still uncommon internationally for LHWs to administer injections to patients. As far as could be ascertained, this is the first study to assess multiple stakeholder perceptions of task-shifting of DOT supervision and administration of intramuscular MDR-TB injections to LHWs in Eswatini. In this study, the multiple stakeholders comprised of representatives from the MOH, donor organisations, professional regulatory institutions, nursing academia, civil society, professional healthcare providers and CTSs in Eswatini. This study elucidates some of the factors that decision-makers should consider when expanding MDR-TB treatment access through task-shifting of DOT supervision and injection administration to LHWs in rural settings.

## Methods

### Setting and design

The Shiselweni region had an estimated population of 204 111 in 2017 [24]. The region has one referral hospital and two health centres supporting 18 smaller PHC clinics that form part of the regional health network managed by the MOH. The referral hospital provides specialised and general health care for the region, while the two health centres, each with an approximately 40-bed capacity, provide medical, surgical and maternal referral care, support and supervision of the PHC clinics. The PHC clinics provide outpatient services to the community and are usually staffed with nurses.

A mixed methods study comprising of a cross-sectional survey using a semi-structured questionnaire with CTSs and a focus group discussion (FGD) with key stakeholders was conducted simultaneously in May 2017. Patients’ perspectives on task-shifting community-based MDR-TB care are analysed in another paper. Throughout this study, the term key stakeholder is used to refer to FGD participants. Data triangulation aided in the comparison and interpretation of the two sets of results.

## Sampling

A purposive sample of 82 out of a study population of 114 CTSs enrolled in community-based MDR-TB management in the Shiselweni region was recruited for participation in the survey. Eligibility criteria required CTSs to have at least one month experience of administering DOT and MDR-TB injections. The study excluded 31 CTSs who had provided community-based MDR-TB care for less than a month and one late withdrawal due to an acute illness.

Participants for the FGD were purposively selected from across diverse national stakeholders based on their potential to generate rich perspectives on the task-shifting approach from the senior positions they occupied in policy-making and advocacy and experience in the fields of HRH and day-to-day practice in MDR-TB management. Participants were drawn from the MOH, donor organisations, professional regulatory institutions, nursing academia, civil society and healthcare providers. Table 1 depicts the FGD participants. Representatives were invited to participate in the FGD in person and via telephone and email. All eligible respondents that were approached agreed to participate in the FGD.

**Table 1:** Summary description of FGD participants by level

Key stakeholder type	*N* = 10	Description
Ministry of Health	3	National-level officials working in healthcare system administration, policy-making, programme development or leadership
Donor partners	2	Individuals working as leaders or managers of international entities providing financial aid or serving as a MDR-TB treatment implementing partner
Professional regulatory institution	1	Representative from the Eswatini Nursing Council, an autonomous body that provides professional guidance and imposes conditions on the practice of nurses
Academia	1	Individual representing an institution providing formal training in nursing
Civil society	1	Non-governmental actors that participate and engage in public health advocacy and policy reform
Healthcare providers	2	Professionally trained medical doctors and nurses working in a health facility providing MDR-TB treatment

### Instrument development and data collection

A semi-structured questionnaire for interviews with CTSs was developed based on literature review and CTS training materials and job descriptions [13–20, 22]. The questionnaire consisted of items measuring CTSs’ demographic details and perceptions on different elements of task-shifting such as awareness, risks and benefits, compensation, retention, and regulation. The content validity of the data collecting tool was assessed by expert opinion. The questionnaire was forward-translated from English to siSwati and back-translated to English before being pre-tested for face validity and understanding in a convenience sample of ten CTSs who were excluded in the main study. The information from the pre-testing was used to revise and improve the clarity of the questions.

CTSs accompanying their MDR-TB patients for their monthly review at the three MDR-TB treating facilities were informed about the research by the community MDR-TB nurse at the end of their consultation. The CTS was then referred to a trained research assistant stationed in a private room within the MDR-TB unit at the health centre. Three research assistants with previous experience in data collection were recruited for the study. The trained interviewers conducted face-to-face interviews in either siSwati or English with CTSs at one of the three rural health centres in the Shiselweni region. The interviews typically lasted between 30 and 45 min.

The FGD was organised topically to verify findings from the interviews with CTSs. The FGD followed a topic guide which included six open-ended exploratory questions (with probes) assessing the following areas: (1) awareness of task-shifting in community-based MDR-TB care; (2) potential risks and benefits of task-shifting; (3) opinion on whether CTSs should be compensated for the tasks they perform; (4) perception on what should be done to retain CTSs; (5) perspectives on the regulation of task-shifting, and (6) acceptability of task-shifting of community-based MDR-TB care to CTSs as a strategy to increase access to MDR-TB treatment.

The FGD was held in a hotel conference room to allow for private conversation without interruption. The first author and one research assistant who assumed the role of note-taker conducted the FGD. Key issues raised during the FGD were summarised on a flip chart to facilitate collective member checking. The researcher used member checking to clarify meanings and generate potentially more valid interpretations. The FGD lasted for an hour and was conducted in English, digitally recorded and transcribed verbatim by research assistants. The first author performed quality checks. A debriefing session between the first author and note-taker was convened after the FGD. The notes served as supplementary documentation and also captured body language and other non-verbal cues used during the FGD.

The researcher visited the potential FGD participants in person 2 weeks prior, sent personalised invitations 1 week before and telephoned each stakeholder a day before the session to remind them. The interaction served to build relationships between the research team and the participants. The facilitator was flexible to adjust to the flow of the discussion, remain open to changes in the FGD guide, remain neutral, and politely enforce ground rules throughout the discussions in order to diplomatically level power dynamics in the FGD. Candid moderation supported by giving everyone a chance to be heard and discouraging participants to interrupt others while they are speaking was used to control dominant individuals.

Participation in the study was voluntary and no rewards were offered for participation. No personal identifying information was collected and findings were reported anonymously using aggregate analysis. Verbal informed consent was obtained from all participants prior to their participation in interviews and audio-recording of the FGD. Participants were informed of their right to discontinue participation permanently or withdraw consent to take part in the study at any point without reprisal. Anonymity of the ten participants in the FGD was achieved through allocation of a coding letter to individual respondent’s data. During the FGD, each participant wore a small card (nametag) displaying their assigned letter. Any personal identifying information revealed during the FGD was removed during transcription. To ensure confidentiality, the digital audio recordings were kept under lock and key by the first author.

### Data analysis

The data was first checked for completeness and consistency by the first author. Data from the questionnaire were double entered into Epi Info 7 and exported to Stata (Version 13.1) for analysis. Standard descriptive statistics were used to summarise the responses.

Qualitative data from the interviews and FGD were analysed using content analysis. First, two coders independently reviewed responses to each open-ended question, identified and grouped similar responses into sub-categories for a sample of ten participants. Second, the coders compared notes and resolved any differences that showed up on their initial sub-categorisation to ensure that they were independent, mutually exclusive and exhaustive. Third, the first author used the consolidated list of generic categories to independently apply coding to the data. The frequency of each of the established categories was calculated. Infrequent categories were collated to form the broader category ‘other’. Fourth, the categories were then grouped under the emerging main themes. Data from the interviews with CTSs was compared with findings from FGD to add breadth and depth to stakeholder perceptions on task-shifting DOT and MDR-TB injection administration to LHWs. Triangulation aided in determining areas where stakeholder perspectives from the two sources were in agreement or divergence.

### Ethical clearance and study authorisation

Ethical approval was obtained from the Scientific and Ethics Committee of Eswatini and the University of the Free State’s Health Sciences Research Ethics Committee (IRB00006240). Authorisation of the study was granted by the NTCP and MSF.

## Results

In this section, we first give a description of the socio-demographic characteristics of the CTSs and FGD participants. We then report the responses given by the CTSs and compare it with reactions of the FGD participants. CTSs’ and FGD participants’ socio-demographic characteristics are described in Table 2. A large majority of CTSs (*n* = 78; 95.1%) were female and 33 (40.2%) were older than 50 years. Most (*n* = 7; 70.0%) key stakeholders had more than ten years of work experience.

**Table 2:** Socio-demographic characteristics of CTSs and key stakeholders

	CTSs, *N* = 82 (%)	Key stakeholders, *N* = 10 (%)
Sex		
Male	4 (4.9)	6 (60.0)
Female	78 (95.1)	4 (40.0)
Age group		
≤ 30 years	10 (12.2)	1 (10.0)
31–40 years	21 (25.6)	2 (20.0)
41–49 years	18 (22.0)	4 (40.0)
≥ 50 years	33 (40.2)	3 (30.0)
Education level		
Primary school or lower	41 (50.0)	0 (0)
Secondary school or higher	41 (50.0)	10 (100.0)
Months administering MDR-TB injections		
1–4 months	17 (20.7)	
> 4 months	65 (79.3)	
Key stakeholder experience in position		
1–5 years		1 (10.0)
6–10 years		2 (20.0)
> 10 years		7 (70.0)

### Level of awareness of task-shifting practices in Eswatini

A total of 78 (95.1%) CTSs considered MDR-TB to be a major public health challenge as shown in Table 3. Over four-fifths (*n* = 70; 85.4%) of the CTSs believed lay community members played an important role in MDR-TB care and the achievement of national and global TB targets. Most FGD participants identified the delegation of counselling, testing services and treatment adherence support to LHWs at PHC clinics offering antiretroviral treatment as examples of on-going task-shifting practices in the country. There was apparent awareness among all key stakeholders of the task-shifting of community-based MDR-TB care to CTSs in the Shiselweni region.

**Table 3:** CTSs’ awareness of task-shifting (*n* = 82)

Statement	*n*	%
MDR-TB is a major public health threat in Eswatini (yes)	78	95.1
Community members should play a role in MDR-TB care (yes)	70	85.4
Are you aware of any task-shifting of responsibilities from professional nurses to lay health workers in relation to medical conditions other than MDR-TB in Eswatini? (yes)	75	91.3
What type of CTS roles do you think is preferable		
Specialist (limited to DOT supervision and administering MDR-TB injections)	14	17.1
Generalist (providing MDR-TB and other PHC needs of the community)	68	82.9

A majority of CTSs (*n* = 68; 82.9%) expressed preference for a ‘generalist’ type of role with a broader mandate not solely limited to MDR-TB care. FGD participants indicated that the community often had a wide range of expectations that fell outside the prescribed responsibilities of CTSs.

*“The community view CTSs as ‘small’ nurses. Some of the CTSs often tell me that they are approached by people looking for medicines to treat fever, diarrhoea or simple first aid.”* [Participant D].

### Potential risks and benefits of task-shifting

Table 4 illustrates potential risks and benefits of task-shifting community-based MDR-TB care identified by CTSs.

**Table 4:** Open-ended responses by CTSs regarding potential risks and benefits of task-shifting community-based MDR-TB care (*n* = 82)

	*N* (%)
Potential risks (*n* = 213)	
Compromised quality of care	60 (73.2)
Malpractice liability fears	40 (48.8)
Poor infection prevention and control	36 (43.9)
Inadequate training	20 (24.9)
Irregular supervision	18 (22.0)
Increased non-adherence to treatment	14 (17.1)
Poor retention of CTSs	10 (12.2)
Power conflict with community MDR-TB nurses	9 (11.0)
Other^a^	6 (7.3)
Potential strategies to mitigate the risks (*n* = 174)	
Appropriate training	69 (84.1)
Regular supportive supervision	40 (48.8)
Simplified instructions and job aids	31 (37.8)
Regulation	23 (28.0)
Improved availability of medical supplies	11 (13.4)
Potential benefits (*n* = 281)	
Increased MDR-TB treatment access	78 (95.1)
Reduced transport-related treatment access barriers	65 (79.3)
Improved adherence to MDR-TB treatment	63 (76.7)
Reduced stigma	32 (39.0)
Improved social status of CTSs	21 (25.6)
Reduced workload for community MDR-TB nurses	15 (18.3)
Increased pool of healthcare workers	7 (8.5)

^a^Other includes disintegrated healthcare system, uncertainty over long-term sustainability and reduced focus on training skilled healthcare workers

CTSs cited suboptimal quality of care (*n* = 60; 73.2%), fears of malpractice litigation (*n* = 40; 48.8%) and poor IPC (*n* = 36; 43.9%) as some of the major risks associated with the task-shifting strategy in the Shiselweni region. Implicitly, these responses potentially represent CTSs’ concerns about inadequacies in the pre-service training and on-going supervision they receive. Not surprisingly, substantial proportions of CTSs suggested improved on-going training (*n* = 69; 84.1%) and supervision (*n* = 40; 48.8%), and simplified instructions and job aids (*n* = 31; 37.8%) as potential strategies to mitigate the risks.

Similarly, key stakeholders representing policy-makers, professional regulatory bodies, academia and civil society in the FGD raised concerns about compromised standards of care and safety risks of shifting invasive clinical procedures such as intramuscular injection administration to LHWs. The absence of a standardised training curriculum for CTSs exacerbated the perceived fear of improper dosing, suboptimal management of adverse events, potential legal implications, transmission of infections through unsafe injection handling and inappropriate community MDR-TB IPC practices among stakeholders. Inadequate oversight and erratic supervision by community MDR-TB nurses and lack of role clarity were other important risks discussed by FGD participants.

Nearly all CTSs (*n* = 78; 95.1%) cited increased access to MDR-TB treatment as the primary advantage of task-shifting. Task-shifting was thought to be helping patients overcome transport-related treatment access barriers (*n* = 65; 79.3%), improving medication adherence (*n* = 63; 76.7%) and reducing stigma (*n* = 32; 39.0%). These results were corroborated by findings from the FGD. Based on their experience of the role of LHWs in HIV programmes, FGD participants acknowledged the potential role of CTSs to facilitate efficient scale-up of MDR-TB treatment especially in rural and remote areas. By enabling the supervision of DOT and administration of MDR-TB injections in the patients’ homes, the task-shifting strategy was perceived to improve patient adherence to treatment and reduce stigma associated with the disease. Furthermore, task-shifting was thought to be helping patients overcome common transport-related treatment access barriers such as long distance travels, cost of transportation and poor road infrastructure that often result in missed or delayed clinic appointments.

*“Task-shifting is allowing MDR-TB patients to receive injections in their homes from CTSs instead of boarding a bus to the clinic daily. In this way, MDR-TB patients also avoid going to the clinic where some people with stigmatising attitudes may not be comfortable to sit in a clinic queue with them due to fear of getting the disease.”* [Participant H].

There were numerous shared views among key stakeholders supporting the delegation of tasks to CTSs such as creating awareness about MDR-TB, referring suspected TB cases to the clinic, supervising DOT and reporting side effects to the community MDR-TB nurse.

### Compensation and retention of CTSs

All CTSs reflected positively on the fact that they received a monetary incentive to cover their own transport when accompanying their MDR-TB patient for review at the nearest clinic. However, some FGD participants—frontline health workers, donor partners and civil society—perceived the monetary incentive to be too little and even in direct contravention of the country’s employment laws. These participants argued that LHWs should be viewed as employees and not volunteers, and hence, should earn a monthly salary equivalent to or above the minimum wage. Conversely, the provision a monthly monetary incentive to CTSs as a general rule was challenged and viewed to be contrary to volunteerism by policy-makers, regulatory authorities and academia.

Over half (*n* = 44; 53.7%) of the CTSs surveyed suggested that they should be compensated because of their perceived high occupational risk of acquiring MDR-TB infection. Key stakeholders iterated that CTSs provided direct care to patients for long periods in the household setting often with poor IPC measures. Participants agreed that the retention of LHWs may be encouraged, or conversely, attenuated by the design and appropriateness of the compensation strategies. Alongside appropriate compensation, participants suggested competency-based pre-service and on-going on-the-job training, adequate supervision, and provision of uniforms, badges and airtime for communicating with the facility-based nurses. Some CTSs suggested that the community MDR-TB nurse who supervised their work should introduce them to the local chiefs and other community leaders for their work to be appreciated.

Almost one-third (*n* = 26; 31.7%) of the CTSs expressed dissatisfaction over the lack of permanent career opportunities in the public health system despite many months of service as entry-level frontline healthcare workers, a suggestion that drew hesitant responses from MOH representatives in the FGD. The participants viewed LHWs as de facto volunteers whose integration into the hierarchy of the health system would be unsustainable.

*“Incorporating CTSs into the public health system and payroll will not be sustainable. The resources are not enough considering the challenges we are facing to improve remuneration of trained doctors and nurses to stop them from leaving for greener pastures.”* [Participant G].

### Regulation of task-shifting

Over a quarter of the CTSs (*n* = 23; 28.0%) indicated that regulatory support would provide a conducive and enabling environment for the task-shifting practice. Almost all FGD participants concurred with this view. Participants identified the need to optimise the standard and quality of care, mitigate potential malpractice liability fears for CTSs, and comply with WHO global recommendations and guidelines for task-shifting as major factors compelling the need to regulate delegation of community-based MDR-TB care to CTSs.

*“Imagine what would happen if patients knew their rights and had the legal knowledge to approach courts and seek redress in case of adverse events due to injections administered by lay people. So regulation will set quality standards and protect CTSs to a certain extent.”* [Participant A].

Stakeholders agreed that the MOH should work closely with relevant professional institutions in developing a competency-based and accredited curriculum for CTS education that is aligned with the country’s National Qualifications Framework. Participants urged the MOH to expedite the adoption of the task-shifting policy that will provide an enabling environment for regulation.

*“Right now the only national policy on task-shifting is still in the draft phase since 2011. Unfortunately, similar to the WHO guidelines on task-shifting, this draft Task Shifting Implementation Framework which should be adopted is biased towards preventive rather than curative services. So delegation of injection administration to lay people may not be adequately covered.”* [Participant B].

### Acceptability of task-shifting

Although generally positive, this question drew a broad range of conflicting opinions about task-shifting, largely stratified by the current job position and day-to-day sphere of practice of the key stakeholders. Table 5 presents some of the verbatim opinions. Most key stakeholders acknowledged that task-shifting in most geographically inaccessible rural areas of Eswatini was driven by necessity and the alternative to this practice would be no MDR-TB care at all.

**Table 5:** Selected participants’ opinions on task-shifting community-based MDR-TB care to CTSs

Supporting views	Opposing views
*Task-shifting is good for the patient especially those too ill or do not have transport money to travel daily to the clinic for injections. I see task-shifting of injection administration to patients’ neighbours as an important part of the so-called patient-centred care.* [Participant C]	*Instead of letting nurses continue throwing their roles to lay people, we should train more professional healthcare workers and ensure they don’t leave for greener pastures.* [Participant F]
*Most patients stay in villages that are difficult to reach with cars. With proper training, CTSs are the only people able to reach these patients. Otherwise rural patients will not get MDR-TB treatment at all. The just need proper guidelines and tools prepared in SiSwati* (local language). [Participant D]	*The use of lay people to give injections is unethical but it is better than nothing. Do you think you can train CTSs to respect the confidentiality of the patient’s medical problems in three days? Giving injections involves exposing and sometimes seeing the intimate anatomy of patients. This takes place in a home setting where there is no privacy. Let us adopt task-shifting, but at the same time, we should address the root causes for shortages in nurses.* [Participant B]
*The delegation of tasks to community health workers is working in HIV. Of course, to use lay people to provide injections is complicated. So the MOH should speed-up the process of putting together a comprehensive policy to guide the use of LHWs.* [Participant H]	*Task-shifting is the only solution though. But I still want to have a look at the curricula for training CTSs. I am convinced it’s unsafe for the patients to receive injections from people other than nurses. Very soon patients will think they are receiving second-class care from CTSs.* [Participant A]
*An adequate number of healthcare workers will always be scarce even if we build nursing schools in every region. The increase in MDR-TB cases makes the situation worse. However, task-shifting to CTSs in Shiselweni improved treatment adherence and MDR-TB treatment cure rates that are important in achieving targets set by the country, WHO and SDGs.* [Participant I]	*In the short-term, we can use task-shifting. Is this approach a magic bullet for problems in our healthcare system? No! To me, this is just a half-baked hurried solution for the poor. A few things need to be done before scaling-up the use of CTSs to provide MDR-TB treatment. Besides, in case of a complication arising from poor injection practice by a CTS, how will the liability be adjudged? Is it the CTS, the trainer, the implementing organisation or the MOH that is liable? Is the training effective enough to teach CTSs infection prevention and safe handling of injections?* [Participant J]

Frontline healthcare workers—doctors and nurses—and donor partners involved in MDR-TB treatment were almost unanimous in reporting positive experiences with CTSs taking care of patients enrolled at their MDR-TB treating facilities. Task-shifting was more acceptable to this group of stakeholders as they were directly confronted in their day-to-day practice by some of the barriers that limit patients from accessing MDR-TB treatment. These participants indicated that CTSs seemed to enjoy performing their tasks, were appreciated by patients and improved treatment adherence.

Policy-makers, and to a lesser extent, regulatory authorities and academia, were less likely to support the use of LHWs to administer intramuscular MDR-TB injections. These stakeholders were sceptical about the effectiveness of the once-off narrowly tailored training provided to CTSs on assuming duties. It was thought that without on-going continuous training, the knowledge and skills of CTSs delivering community-based MDR-TB care was likely to fall-off over time, raising further patient safety concerns. Regulatory authority and academia representatives expressed dissatisfaction about the increased attention paid to task-shifting as a panacea to the critical shortage of professional HRH in Eswatini, while neglecting long-standing challenges in training, recruitment and retention of professional healthcare workers, particularly in rural areas.

## Discussion

This study not only extends the growing literature on task-shifting, but also contributes new insights critical for evidence-informed policy-making and practice regarding the delegation of DOT and highly differentiated clinical tasks such as administration of MDR-TB injection to LHWs in resource-limited settings like Eswatini. This study highlights considerable contextual differences that influence the perceptions on and acceptability of task-shifting DOT and MDR-TB injection administration to LHWs by stakeholders, and further illuminates substantial gaps in the current strategy that warrant attention. There was considerable support for and consensus among key stakeholders around the acceptability of task-shifting practices in community MDR-TB management, albeit with some reservations and apprehensions.

Consistent with results from previous studies, findings from this study highlight systemic concerns that may impede the task-shifting practice, including underinvestment in training, erratic supervision, lack of appropriate compensation, and absence of regulation of the practice [17–20, 22, 25, 26]. Nevertheless, findings from the interviews and FGDs identified the following conditions as important enablers for the acceptance and formalisation of the practice in Eswatini: adequate pre-service and continuous accredited training, appropriately tailored protocols and job aids, on-going structured supportive supervision, recognition, compensation, and regulation [13, 15, 17–20, 22, 26].

Participants widely viewed LHWs as essential frontline HRH critical for the successful scaling-up of MDR-TB treatment access and achievement of MDR-TB control targets outlined in the National TB Strategic Plan, End TB Strategy and SDGs. In this study, a majority of participants preferred ‘generalist’ type of LHWs with a wider scope of practice and mandate aimed at providing contextually appropriate integrated basic PHC needs of the community. These findings highlight the tension that exists between the current LHWs’ disease-specific scope of work and the community’s broad preventive and curative PHC needs and expectations [27]. LHWs could leverage on their physical accessibility and understanding of local issues—shared language, culture and strong connection with community members—to extend the reach of healthcare services to remote settings where HRH are scarce [28, 29].

Similar to evidence from previous studies [26, 30, 31], CTSs preferred to be paid, but barring that, also appreciated non-monetary incentives. Nevertheless, policy-makers and regulatory authorities commented that providing these incentives may have subtle pitfalls of undermining the long-term sustainability of the task-shifting approach. Many of the recommendations proposed by CTSs and key stakeholders suggest that strong social networks, social cohesion and support systems at the community level that validate the roles and responsibilities of LHWs play an important role in improving their retention [26, 30, 31].

By delegating clinical tasks traditionally restricted to professional nurses to LHWs, the status and expectation of CTSs is likely to change and challenge traditional hierarchies [18, 22]. The retention of CTSs could be strengthened by paying attention to opportunities for their financial independence and social recognition [26, 30]. These factors coupled with inputs by the programme such as access to competency-based training and certification, supportive supervision, adequate logistical support, and opportunities for career progression and being integrated into the formal health system determine whether or not CTSs continue in their role [13, 22, 26, 30, 31]. This finding correlates favourably with the WHO’s Global Strategy on Human Resources for Health 2030, that emphasises the need to design appropriate strategies and incentives to retain LHWs and optimise workforce capacity [14]. Although this research limited its scope to assessing factors affecting retention of CTSs, the findings provide a base for future studies exploring potential influences of LHW attrition in Eswatini.

In this study, concerns about the clinical quality of care, patient safety and malpractice liability fears were cited as important reasons compelling the need to regulate task-shifting by both CTSs and key stakeholders, particularly from participants representing the MOH, professional regulatory bodies and training institutions. These results appear to validate similar disquiets expressed in Mozambique [20] and Uganda [17–19]. Key stakeholders in the current study proposed that the MOH, professional regulatory bodies and training institutions work in close collaboration to create a supportive regulatory environment for task-shifting.

Similar to recommendations from global research on task-shifting, participants suggested a number of elements that could facilitate the regulation of task-shifting, broadly comprising of: (1) expediting the formalisation of the country’s task-shifting policy that was still in the draft phase; (2) developing minimum requisite literacy and numeracy skills for CTSs; (3) standardising pre-service and on-going curricula design; (4) clarifying roles and extent of scope-of-practice; and (5) establishing a supportive conducive environment including supervision and appropriate recognition [15, 25, 30, 32]. Implicitly, in this study, there was minimal compliance to the WHO recommendations and guidelines on task shifting that propose the need for consultation, national endorsement, an enabling regulatory framework and quality assurance mechanisms including standardised training, certification and regular supportive supervision important in optimising quality of care [13].

Frontline healthcare workers and donor partners that encountered MDR-TB patients in their day-to-day practice were more supportive of the task-shifting approach compared to policy-makers and other stakeholders. Those supportive of the strategy went beyond viewing task-shifting simplistically as a way of shifting responsibilities to cheaper and lower skilled LHWs. They justified task-shifting on various grounds including improved medication adherence, economic considerations and as an appropriate response to frontline TB HRH shortages, among others. This is consistent with results from previous studies [17, 20, 22].

Dissenters to the strategy described task-shifting with terms such as “unethical”, “unsafe”, “second-class care”, and “a half-baked hurried solution for the poor” suggesting that the concept was not fully understood by all stakeholders. The divergent views on task-shifting resonate globally. Although frontline healthcare workers in South Africa [33], Mozambique [20] and Tanzania [34] were found to support task-shifting, policy-makers in Uganda [19] opposed it. Yet, it has been conclusively shown in Uganda that without adequate support and endorsement from policy-makers, task-shifting may still occur but in a disorganised manner, potentially compromising the standard of care [19].

Some key stakeholders—professional regulatory authorities and academia—in this study believed that the task-shifting strategy was overshadowing long-standing challenges with training of new and retention of existing professional HRH, substantiating previous findings [19, 34]. In Tanzania, respondents viewed task-shifting as a short-term solution whilst the government considered more permanent and sustainable alternatives to address the shortage of healthcare workers [34].

## Limitations of the study

With this study mostly founded on self-reported perceptions of task-shifting community-based MDR-TB care to LHWs, it is possible that participants’ responses may reflect socially desirable answers rather than their true views and experiences of task-shifting. However, this was partially countered by assuring respondents of the anonymity of the questionnaires and FGD, and that findings would be reported using aggregate analysis and that the outcomes of the study were not linked to CTSs’ job security. Due to practical constraints, this study excluded the participation of family members and other caregivers who could have provided diverse and instructive opinions about task-shifting community-based MDR-TB care to CTSs. Future studies could seek to understand their opinions on the practice.

## Conclusion

Taken together, stakeholders generally accepted the delegation of DOT supervision and administration of intramuscular MDR-TB injections to LHWs as a strategy to increase access to treatment, albeit with some apprehension. Findings from this study suggest that task-shifting is considered as an acceptable interim measure that can be implemented alongside other strategies to train, attract and retain adequate numbers of professional healthcare workers in Eswatini.

The absence of explicit holistic policy guidance and sufficient support from policy-makers for task-shifting at national level undermine the acceptance and the potential for scaling-up the approach in Eswatini. To address some of the apprehension and ambivalence about expanding access to MDR-TB services through task-shifting, attention should be paid to important aspects such as competence-based training, certification and accreditation, adequate supportive on-the-job supervision, recognition, compensation, and expediting policy and regulatory support for LHWs. Future studies should explore the underlying reasons for the differences in opinions between policy-makers and professional healthcare workers and the extent to which this may undermine the successful formalisation of task-shifting community-based MDR-TB care to LHWs in Eswatini.

## Data Availability

The data analysed during this study are not publicly available as individual privacy would otherwise be compromised. The data that support the findings of this study are available on request from the corresponding author EP.

## Abbreviations

CTS: Community treatment supporter
DOT: Directly observed treatment
FGD: Focus group discussion
HRH: Human resources for health
LHW: Lay health worker
MOH: Ministry of Health
MSF: Médecins Sans Frontières
MDR-TB: Multidrug-resistant tuberculosis
NTCP: National Tuberculosis Control Programme
PHC: Primary health care
SDG: Sustainable Development Goal
TB: Tuberculosis
WHO: World Health Organization
